# An Overview of Biomembrane Functions in Plant Responses to High-Temperature Stress

**DOI:** 10.3389/fpls.2018.00915

**Published:** 2018-07-03

**Authors:** Yue Niu, Yun Xiang

**Affiliations:** MOE Key Laboratory of Cell Activities and Stress Adaptations, School of Life Sciences, Lanzhou University, Lanzhou, China

**Keywords:** high temperature, plasma membrane, ER unfolded protein response, thylakoid membrane, heat-inducible pathway, membrane stress responses

## Abstract

Biological membranes are highly ordered structures consisting of mosaics of lipids and proteins. Elevated temperatures can directly and effectively change the properties of these membranes, including their fluidity and permeability, through a holistic effect that involves changes in the lipid composition and/or interactions between lipids and specific membrane proteins. Ultimately, high temperatures can alter microdomain remodeling and instantaneously relay ambient cues to downstream signaling pathways. Thus, dynamic membrane regulation not only helps cells perceive temperature changes but also participates in intracellular responses and determines a cell’s fate. Moreover, due to the specific distribution of extra- and endomembrane elements, the plasma membrane (PM) and membranous organelles are individually responsible for distinct developmental events during plant adaptation to heat stress. This review describes recent studies that focused on the roles of various components that can alter the physical state of the plasma and thylakoid membranes as well as the crucial signaling pathways initiated through the membrane system, encompassing both endomembranes and membranous organelles in the context of heat stress responses.

## Introduction

The Fifth Assessment Report of the IPCC (Intergovernmental Panel on Climate Change [IPCC], 2014a) showed that the increase in the global average surface temperature from 1880 to 2012 reached 0.85°C, and the last three decades in particular were persistently warmer than any preceding decades. Moreover, the report predicted that the extreme annual daily maximum temperature would be elevated by approximately 1–3°C by the mid-21st century (Meehl and Tebaldi, 2004; Intergovernmental Panel on Climate Change [IPCC], 2014a). Although warming has been observed to benefit crop production in some high-latitude regions, the increasing threat of climate change has had negative impacts on crop yields over a wide range of areas, and the detrimental effects are expected to be aggravated in the next century (Intergovernmental Panel on Climate Change [IPCC], 2014b). In addition, secondary disasters that accompany high temperatures, such as drought and soil salinization, could trigger dramatic crop loss worldwide and even lead to global food shortages in the future. In fact, regardless of the climatological extremes, plants, being sessile, are inevitably exposed to a wide range of temperature stresses, including daily and/or seasonal temperature fluctuations that may reach dozens of degrees. Each plant species naturally exhibits its own optimal temperature range for growth and reproduction, and thus, extreme variations can rapidly exert a thermodynamic influence on the intracellular macromolecules (nucleic acids and proteins) and substructures of plant cells (Ruelland and Zachowski, 2010). For instance, a temperature of 5°C above optimal growing conditions can induce a series of changes in the organization of cell compartments, including the organelles and cytoskeleton, as well as the membrane microdomains necessary for plant survival at high temperatures (Weis and Berry, 1988; Guy, 1999; Bita and Gerats, 2013). Plants have evolved different adaptation mechanisms to withstand adverse conditions for optimal growth, the most common of which involve the activation of stress-induced gene expression related to the homeostatic adjustment of plant metabolism and development (Iba, 2002; Shinozaki et al., 2015). Therefore, breeding efforts focused on the transformation of these genes as supported by genetic approaches have been employed to efficiently improve plant adaptability to extreme temperatures (Grover et al., 2000; Sharkey, 2000). In particular, heat shock transcription factors and heat shock proteins (HSFs/HSPs) have been demonstrated to constitute a master regulatory pathway involved in the response to heat stress based on half a century of research, and some of these genes have since been applied in sustainable agriculture (Ritossa, 1962; Scharf et al., 2012). Nevertheless, in considering the mechanisms underlying the high-temperature stress response, one should not ignore how plants first quickly perceive and translate the stress into intracellular response signaling. In this respect, cell membranes, which constitute the barrier to the external environment and separate the cytosol into several microcompartments, are well suited for sensing stress and providing a “crosstalk" interface. Subtle alterations in lipids may affect various properties of the membrane, including its fluidity, thickness, permeability, and packing, and thus affect the clustering of important membrane proteins, which are sensitive to ambient conditions (Escribá et al., 2008). However, unlike in animal cells, membrane structures in plant cells possessing a cell wall may display an enormous and specific variety of components and functions (Malinsky et al., 2013). Combined with the diversity of plant responses to adverse environments, this complexity is such that the majority of the underlying thermotolerance mechanisms in plants remain unclear. The various challenges remaining in this field will be addressed in the following open questions.

In this review, we provide an overview of the currently known factors and/or mechanisms that can affect the states and properties of the plasma membrane (PM) and thylakoid membrane as well as the endoplasmic reticulum system (ER) in responses to and in tolerance of high-temperature stress in land plants (largely in reference to *Arabidopsis thaliana*). Some critical perspectives on the roles of membranes in these processes will also be presented, and we will discuss membrane-stress responses under heat stress within different plant taxa, including several psychrophiles, mesophiles, and thermophiles in the plant kingdom (as classified by Levitt, 1980; Zrobek-Sokolnik, 2012; **Table 1**).

**Table 1:** Membrane-stress responses under heat stress within different plant taxa.

Species	Distribution	Optimum temperature	Heat stress	Membrane-associated tolerance mechanisms	Reference
*Deschampsia antarctica* *Colobanthus quitensis*	The west coast of Antarctica	10°C 12–14°C	>22°C >26°C	The rates of whole-canopy net photosynthesis (Pn) are sharply decreased under high temperature; can still keep 30% of the maximal rate of Pn when temperature close to 0°C.	Xiong et al., 2000
Green alga (*Chlamydomonas reinhardtii*)	World-wide (soil)	24°C	>37°C	Rapidly increasing glycerophosphoglycerol (GPG, splitted from PG by PA/lipid acyl hydrolase); Increasing in saturated FAs and TAGs with polyunsaturated FAs accumulated within lipid bodies; Rapid arrest of CO_2_ fixation for NADPH and ATP in *de novo* synthesis of saturated FAs; LHCII uncoupling and reactivating CO_2_ fixation in long term HS; Decrease of PG and SQDG may restrain photosystem assembly; Chaperones are rapidly accumulated in cytosol, plastid, mitochondrion and ER.	Lamosa et al., 2000; Pröschold et al., 2005; Merchant et al., 2006; Hemme et al., 2014; Schroda et al., 2015
Rice (*Oryza sativa*)	Tropical and subtropical regions of South and Southeast Asia (China, Japan, Philippines etc.)	Day/night temperature 30°C/25°C	>35°C; >40°C (sufficient irrigation water)	The homologs involved in heat stress response in *Arabidopsis* mostly can be identified in rice. e.g., Some Rice RBOHs (NADPH oxidases, OsNoxs) are indicated in heat-induced ROS signaling; The oscillation of Ca^2^+ signature can induce the rice CaM1 -1 isoform to transmit the HS signal to sHSPC/N; Rice PEP-associated protein (WLP2) can protect chloroplast development under heat stress via maintaining the redox balance and regulating the expression of PEP-encoded genes.	Wu and Jinn, 2012; Wang et al., 2013; Jagadish et al., 2015; Lv et al., 2017
New Rice for Africa line (NERICAs L-44 (NL-44)) New Rice for Africa line (NERICAs 22 (N22))	Field experiment in India, Philippines	28°C	>38°C	The cultivars possess high photosynthesis, scavenging enzyme activities and membrane stability as well as low ROS products.	Bahuguna et al., 2015
Wheat (*Triticum aestivum*)	Widely cultivated in temperate regions	18–25°C	>32°C	An increased ratio of DGDG to MGDG, especially high levels of saturation of DGDG can enhance the thermostability of thylakoid membrane; Low expression of electrolyte leakage from leaf cells can maintain membrane thermostability; Several genes may simultaneously control the membrane stability under heat stress.	Mullarkey and Jones, 2000; Cossani and Reynolds, 2012; Dhanda and Munjal, 2012
*Arabis paniculata*	Alpine region	22°C	>38°C	The degree of lipid unsaturation is rapidly decreased; and higher level of HSPs is induced and maintained.	Tang et al., 2016
*Dichanthelium lanuginosum*	Geothermally heated environments (North America)	35°–41°C	>45°C	The plant has higher shoot fresh water under high temperature; and the cells express low molecular weight HSPs and thermostable enzymes.	Stout et al., 1997

## The PM Acts as Both Thermometer and Thermostat to Perceive and React to High Temperatures

### PM Composition and Microdomain

Membranes serve as a selectively permeable barrier, and they are primarily composed of proteins and lipids in moving mosaics. Lipids with a polar head group and diverse types of long hydrophobic tails spontaneously form two leaflets in aqueous environments due to their amphipathic properties. Lipid species can be divided into three primary classes by chemical structure, namely, glycerolipids, sphingolipids and sterols (Enrique Gomez et al., 2017). Among the abundant glycerolipids, phospholipids predominantly occur in the PM and mitochondrial envelope, while galactolipids primarily constitute the thylakoid membrane (Dubots et al., 2012). Phospholipids contain two fatty acyl chains and a variable polar head group assembled to the glycerol backbone. Although phospholipids are usually sorted by their polar head groups, the fatty acid moieties greatly control their physicochemical properties (Escribá et al., 2008). The number and position of C–C bonds within hydrophobic acyl tails and the length of the carbon chains are thought to be key factors in determining the membrane fluidity (Fujimoto and Parmryd, 2017). Moreover, the lipid composition of each leaflet reflects another membrane property (asymmetry). Lipids are selectively embedded in membrane bilayers; phosphatidylcholine (PC), glycolipids and sphingomyelin are predominantly enriched on the exoplasmic face of the PM and the lumenal side of internal organelles, while phosphatidylserine (PS), phosphatidylethanolamine (PE), and phosphatidylinositol (PI) are primarily found on the cytoplasmic side; cholesterol shows preferential accumulation on the exoplasmic leaflet, although it can move freely between the two leaflets (Lenoir et al., 2007; Andersen et al., 2016). Additionally, some minor phospholipids, such as phosphatidic acid (PA), phosphatidylinositol-4-monophosphate (PIP) and phosphatidylinositol-4,5-biphosphate (PIP_2_), are also enriched on the cytoplasmic leaflet (Gascard et al., 1991).

**FIGURE 1:**
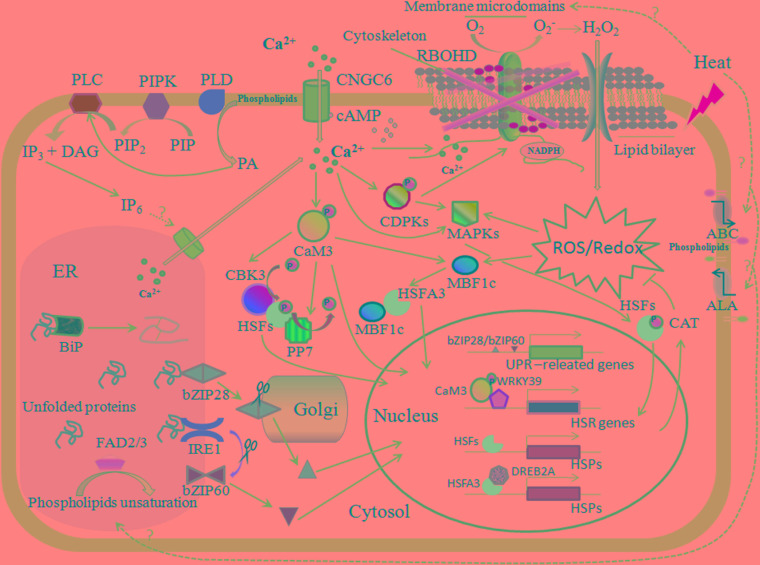
Plasma membrane (PM) and ER are involved in the plant heat stress response. Heat stress alters the physical state of membranes and affects membrane proteins as well as the membrane structure; for example, membrane microdomains can be subtly changed, which induces the clustering of signaling proteins correspondingly to make adjustments. The PM-located RBOHD is embraced within and regulated by the special membrane region; the calcium-permeable channel CNGC6, which is activated by cyclic adenosine monophosphate (cAMP), primarily mediates the calcium influx; and lipid translocases ABC and ALA cope with structural lipid flipping and/or flopping, resulting in changes to the membrane stability. Membrane-associated PIPK, PLD, and PLC perceive changes in the states of membranes and trigger the lipid-signaling molecules PIP_2_, PA, and IP_3_, respectively, and when phosphorylated to IP_6_, IP_3_ can mediate the calcium release from the ER. The burst of cytosolic calcium can also activate the RBOHD, which induces a rapid increase in hydrogen peroxide (H_2_O_2_) and triggers the ROS/redox signaling pathway, including MAPK-HSFs and MBF1c-HSFA3-DREB2A, during the heat stress response (HSR). MAPK-HSFs can regulate the expression of CAT, conversely controlling the level of ROS; the phosphorylation of calcium-dependent protein kinase (CDPK) also activates RBOHD. Calcium binding to CaM3 evokes CBK3/PP7 and the transcription factors of the HSR, such as HSFs and WRKY39. The heat sensors involved in the ER-UPR are comprised of bZIP28 (spliced in Golgi) and IRE1 (splicing *bZIP60* mRNA), and ER-UPR also requires chaperone BiP binding to unfold protein for correct folding. The FAD2 and FAD3 in the ER are important components in the control of membrane fluidity during the HSR.

The fluid mosaic hypothesis proposed by Singer and Nicolson (1972) indicated that lipids formed a discontinuous, fluid bilayer in which proteins were embedded via specific interactions with lipids (). Today, evidence regarding the structure and function of membranes inspires us to consider the biomembrane as a more complex and exquisite device (Vereb et al., 2003; Cacas et al., 2012; Konrad and Ott, 2015). Different regions of membranes with defined lipids and clusters of proteins are not always equal, forming discrete platforms with varying sizes and traits in the plane of the membrane (which are denominated as membrane microdomains) (Karnovsky et al., 1982; Thompson and Tillack, 1985; Simons and Ikonen, 1997; Simons and Toomre, 2000; Lillemeier et al., 2006; **Figure 1**). Historically, lipid rafts enriched in sphingolipids, sterols and glycosylphosphatidylinositol (GPI)-anchored proteins were thought to be equivalent to membrane microdomains or detergent-insoluble membranes (treated with 1% Triton X-100 at 4°C; Brown and Rose, 1992; Cacas et al., 2012; Malinsky et al., 2013). Over the past decade, this idea has come to be considered controversial, if all of the above concepts reflect the authentic membrane substructure (Malinsky et al., 2013). With the development of experimental approaches, the microdomains of membranes can now be distinguished at multiple scales (reviewed by Konrad and Ott, 2015). Nanoscale domains based on lipid rafts (2–100 nm) are incorporated into larger microdomains (e.g., raft platforms) via the interactions between specific lipids and proteins; both cytoskeleton and cell wall components prop up the largest units, which are denominated as the membrane compartments (40–300 nm), depending on the cytoskeletal and cell wall restriction of the lateral diffusion of membrane proteins (Kusumi et al., 2005, 2011, 2012; Jacobson et al., 2007; Martinière et al., 2012). In contrast to lipid rafts with a highly dynamic nature in mammalian cells, special lateral membrane compartments in plant cells are more stable (Cacas et al., 2012; Malinsky et al., 2013). Substantial evidence supports the notion that the microdomain acts as a harbor in which cellular signaling is clustered by the interactions between membrane components (including previously isolated channels, receptors, other signaling complexes and specific lipids) during many physiological processes, such as biotic and abiotic stress responses, membrane transport and polarized plant growth (Laude and Prior, 2004; Lingwood and Simons, 2010; Malinsky et al., 2013; Jarsch et al., 2014; Wang et al., 2018). An ROS-generating enzyme known as respiratory burst oxidase homolog D (RBOHD) has been suggested to localize to membrane microdomains (Lherminier et al., 2009; Hao et al., 2014; **Figure 1**) and could be activated by a heat-induced cytosolic calcium increase to immediately produce an oxidative burst (Suzuki et al., 2011). However, numerous microdomain-associated proteins remain to be elucidated in plants. Notably, increasing evidence from mammals has illustrated that several HSPs localize to PM rafts via interactions with specific lipids (reviewed by Escribá et al., 2008). These HSPs include abundant HSP70, HSP90 and other small HSPs (e.g., HSP27), the functions of which have been suggested to include the recruitment of signaling proteins to the PM, the folding of membrane proteins, assisting polypeptides in translocation across the membrane bilayer, and rapidly remodeling and protecting the PM under cellular stresses (Arispe et al., 2002; Shah et al., 2002; Vega and De Maio, 2005; Vigh et al., 2005, 2007b). Although the mechanism controlling the interactions between HSPs and membrane lipids and proteins is less well known, it is speculated that the structure of lipid rafts is crucial for HSPs to perform their individual functions in cell responses (Escribá et al., 2008). Whether HSPs in plants exhibit similar behaviors at PM microdomains has not yet been explored.

### Lipid Saturation and Desaturases

Plants that survive under extreme temperatures must first maintain constant membrane fluidity and integrity, which requires dynamic changes in the membrane composition. Early studies have shown that the ratio of saturated and unsaturated fatty acids differs greatly between lipid species and tissues (Yabuuchi and O’Brien, 1968; Wood and Harlow, 1969). In addition, the saturated and monounsaturated fatty acid contents increase in response to elevated temperatures, while the proportion of polyunsaturated fatty acids can be increased by a sudden drop in temperature (Wallis and Browse, 2002; Penfield, 2008). Temperature is the most important contributing factor in regulating the unsaturation degree of fatty acid chains, and thus, it has a large impact on the properties of membrane bilayers (Ruelland and Zachowski, 2010; Furt et al., 2011). The numbers and sites of double bonds within fatty acid chains are modified by desaturases via an oxygen-dependent reaction (Aguilar and de Mendoza, 2006). In plants, the fatty acid desaturase (*FAD*) family, which plays an important role in adaptation to high-temperature stress, has been studied extensively. Among the eight members of the *Arabidopsis FAD* family (*FAD1* to *FAD8*), FAD2 and FAD3 are located in the ER membrane, while the others are plasmid-specific desaturases (Browse and Somerville, 1991; Heinz, 1993; **Figure 1**). Although the synthesis and unsaturation of the major lipids are performed in the chloroplasts or ER in higher plants, acyl chain modifications of lipids embedded in the PM rely on regulation by various desaturases and on lipid transfer (Wallis and Browse, 2002; Singer et al., 2016). Ambient stresses, such as temperature fluctuations, are perceived by the PM and/or membranous organelles and can affect FAD activities at both the transcriptional and post-transcriptional levels, thereby altering the membrane fluidity (Singer et al., 2016). Recently, increasing evidence has shown that FAD2 and FAD3 are unstable and less enriched at elevated temperatures (Dyer and Mullen, 2001; Sánchez-García et al., 2004; Tang et al., 2005; O’Quin et al., 2010; Khuu et al., 2011). The seedlings of the *fad2* mutant are unable to elongate their stems at 12°C and die at 6°C (Miquel et al., 1993); similarly, the lethal temperature for *fad1* is 2°C (Wu et al., 1997). Interestingly, the dwarf *fad2* plant has been found to exhibit enhanced reactive oxygen species (ROS) and salicylic acid (SA) signaling, which are considered to regulate the heat stress response (Kachroo et al., 2004; Larkindale et al., 2005). Additionally, other *fad* mutants, such as *fad5*, show higher growth rates depending on the thermal conditions. FAD3, FAD7, and FAD8 have all been demonstrated to confer high-temperature instability (Horiguchi et al., 2000; Matsuda et al., 2005), and three transgenic lines have exhibited higher levels of trienoic fatty acids in response to extreme temperatures (Kodama et al., 1995; Zhang et al., 2005; Penfield, 2008). In fact, the varying amounts of unsaturated fatty acids incorporated into the major glycerolipids in *fad* mutants alter the composition and properties of their membranes, which may be the reason why their cells can sense and react to high temperatures (Wallis and Browse, 2002). Confusingly, however, the mechanism through which FADs rapidly react to sudden temperature fluctuations and upstream factors with temperature sensitivity to regulate the activities of FADs remains unclear in plants.

### Membrane Asymmetry and Lipid Translocases

It is not only the chemical constitution of lipid membrane bilayers that is closely associated with the temperature; the multiple types of molecular motion by lipids (including rotation around their own axes, lateral diffusion within a monolayer and flip-flopping between the two monolayers) involved in the physical properties of the membrane are also highly temperature dependent (Ruelland and Zachowski, 2010). The transbilayer translocation of lipids is crucial for maintaining lipid asymmetry, which is necessary for membrane stability and the production of transport vesicles; translocation is also related to the demands of cell growth as well as cellular responses to physiological stresses (Pomorski and Menon, 2016). For instance, PS acts as a common type of phospholipid that is inserted in the membranes, and the flipping of the PS under stress is considered to be an important “eat-me” signal for macrophages in eukaryotes (Segawa et al., 2011, 2014). In general, the asymmetrical distribution of lipids can be achieved through the actions of three protein families, scramblases, ABC transporters and P_4_-ATPases, which are found in plants and other organisms. The first group of proteins catalyzes the ATP-free bidirectional movement of lipids, whereas most ABC transporters shift lipids from the cytoplasmic to the exoplasmic leaflet (flopping), and P_4_-ATPases do just the opposite (flipping) (Pomorski and Menon, 2016; **Figure 1**). The implications of scramblases in abiotic stress responses in the plant kingdom remain unclear, but there are a number of reports on the involvement of the other two types of floppase/flippase in responding to environmental challenges, including extreme temperatures. ABC transporters in *A. thaliana* belong to a large protein family with 129 members. Among these transporters, a previous study showed that reducing the functions of plasmid-specific ABC proteins TGD1, 2, 3, and 4 can effectively suppress the low-temperature-induced phenotype of the *vte2* mutant (Song et al., 2010). Earlier research indicated that the transcription of a homolog (*TUR2*) of the yeast ABC transporter gene *PDR5* was induced by low temperatures in *Spirodela polyrrhiza* (Smart and Fleming, 1996). Li et al. (2011) found that the expression of ABC1 was specifically induced under a combination of saline and heat shock stresses in *Suaeda salsa*. Moreover, a subsequent report suggested that similar to other abiotic-stress-related genes, ABC transporters were abundantly enriched during cold acclimation in the grapevine *Vitis amurensis* (Wu et al., 2014). Likewise, 12 P_4_-ATPases in the *Arabidopsis* genome designated ALA1 to ALA12 were shown to perform inward lipid translocation (Axelsen and Palmgren, 1998), and the evidence obtained to date demonstrates that the functions of the proteins in this group are all affected to varying degrees by temperature fluctuations. For instance, ALA1 was shown to play a crucial role in chilling tolerance (Gomès et al., 2000), while ALA3 regulates vegetative and reproductive growth in a temperature-dependent manner (McDowell et al., 2013). ALA6 and ALA7 determine the fitness of pollen by regulating adaptations to daily temperature swings (McDowell et al., 2015), and ALA6 has been verified to directly participate in thermotolerance (Niu et al., 2017). In addition, a recent study suggested that ALA10 contributed to regulating the activities of FAD2/FAD3 to control galactolipid synthesis in leaf development, and this positive regulation is susceptible to chilling stress (Botella et al., 2016). Nevertheless, solid evidence for the involvement of lipid translocation in temperature sensing has yet to be obtained.

### Heat-Induced PM Stress Perception

Temperature is a physical cue, and it directly alters the fluidity of biomembranes and the remodeling of membrane microdomains. This may have comprehensive effects not only on the membrane composition but also on specific interactions between lipids and proteins such as ion channels and kinases, subsequently altering the conformation and function of proteins and triggering particular signal transduction pathways (Plieth, 1999; Vigh et al., 2007a; **Figure 1**). Cells remodel and reestablish membrane fluidity to cope with a change in the ambient temperature via the real-time control of lipid saturation and fatty acid length (Schroda et al., 2015). Above all, subtle changes in membrane fluidity should be promptly perceived by PM sensors. Although studies have indicated that there are several ion channels that function as temperature sensors in animal cells, such as the transient receptor potential cation channel subfamily V (TRPV) and cyclic nucleotide-gated channels (CNGCs; Ramot et al., 2008; Yao et al., 2011), the PM thermosensor in plants long remained unidentified until the existence of primary sensors for heat stress was revealed in the PM of *Physcomitrella patens* (Saidi et al., 2009, 2011). However, the membrane fluidizer benzyl alcohol can mimic the effects of high temperatures on the PM calcium-dependent heat shock response (HSR); conversely, the rigidifying agent dimethyl sulfoxide can decrease the heat-activated expression of HSPs (Saidi et al., 2005, 2009; Suri and Dhindsa, 2008), which also supports the conclusion that heat-mediated calcium channels are probably regulated by membrane fluidity (Saidi et al., 2009, 2010). Further evidence of the role of these calcium channels as “thermosensors” in plants is needed. In particular, it is poorly understood how these calcium channels perceive and distinguish among different types of high-temperature stress (e.g., basal heat shock, warm priming/acclimation before heat shock, gradual temperature increases, hot-day/cold-night, or short-term and long-term heat stress).

### Heat-Induced Second Messengers and Signal Cascades

One of the earliest identified plant responses to elevated temperatures was a specific transient Ca^2+^ influx from the extracellular matrix to the cytoplasm (Gong et al., 1998; Liu et al., 2006). The effective suppression of the HSR pathway in higher plants by both calcium chelators and channel blockers suggested that an inward flux of Ca^2+^ and the ensuing signaling could play critical roles in the heat stress response (Braam, 1992; Larkindale and Knight, 2002; Li et al., 2004; Suri and Dhindsa, 2008). The presence of a calcium spike within seconds of thermal treatment in most model plants strongly indicates that heat-sensitive calcium-permeable channels are present in the PM and can transiently open or close during the thermotolerance process (Gong et al., 1998; Liu et al., 2006; Saidi et al., 2009; Wu and Jinn, 2010). Over 40 putative calcium channels located in the PM in the *A. thaliana* genome are regarded as the candidate heat sensors (Ward et al., 2009). Gao et al. (2012) indicated that cyclic nucleotide-gated ion channel 6 (CNGC6) might mediate the heat-activated Ca^2+^ influx and the expression of *HSPs* in *A. thaliana* during plant adaptive responses to heat (**Figure 1**). Additionally, CNGC6 was also activated by cytosolic cyclic adenosine monophosphate (cAMP), the concentration of which could be rapidly increased within 2 min after heat shock. The binding of free Ca^2+^ ions to calmodulins (CaMs) has been demonstrated to trigger PM calcium signal cascades, which can activate the HSF and HSP networks (von Koskull-Döring et al., 2007). In *A. thaliana*, AtCaM3 is primarily involved in the activation of transcription factors comprising HSFs, multiprotein bridging factor 1c (MBF1c), WRKY DNA-binding domain superfamily protein (WRKY39) and dehydration-responsive element binding protein (DREB) (Li et al., 2010; Liu et al., 2011; Suzuki et al., 2011; **Figure 1**). MBF1c has been demonstrated to regulate the expression of over 30 heat-related transcripts, including HSFB2a and HSFB2b as well as DREB2A, which is upstream of HSFA3 (Schramm et al., 2008; Suzuki et al., 2011). WRKY39 facilitates the jasmonic acid and SA signaling pathways under heat stress (Li et al., 2010). With respect to the HSFs and the HSF-HSP signaling pathway, the numerous efforts aimed at understanding these proteins began as early as 1960s and 1970s and will therefore not be addressed in this review (see reviews from Baniwal et al., 2004; Kotak et al., 2007; von Koskull-Döring et al., 2007; Saidi et al., 2011). AtCaM3 binds to the *Arabidopsis* CaM-binding protein kinase 3 (AtCBK3) to promote the phosphorylation of HSFA1a via the activation of a heat-induced transient Ca^2+^ increase (Liu et al., 2008; Saidi et al., 2011), while calmodulin-dependent phosphatase 7 (PP7) mediates the dephosphorylation of HSFA1a in a Ca^2+^-dependent manner (Liu et al., 2007a; Bokszczanin et al., 2013).

Hydrogen peroxide (H_2_O_2_) is considered to be the other crucial second messenger when plants are exposed to elevated temperatures, because a rapid accumulation of H_2_O_2_ can occur within several minutes (Konigshofer et al., 2008). The PM-located protein RBOHD is involved in the generation of H_2_O_2_ and is directly activated by an increase in cytosolic calcium and/or the phosphorylation of a calcium-dependent protein kinase (CDPK; Suzuki et al., 2011; **Figure 1**). The downstream signal cascades of RBOHD also comprise certain HSFs, mitogen-activated protein kinases (MAPKs) and MBF1c, which are involved in HSR (Mittler et al., 2004, Miller et al., 2009; Suzuki et al., 2011; **Figure 1**). For example, HSFA4a might be a sensor of the H_2_O_2_ signaling, while HSFA5 serves as a repressor of this pathway (Miller and Mittler, 2006; Baniwal et al., 2007). MAPKs can affect the expression and activity of catalase (CAT) to control the ROS levels while conversely regulating the expression of RBOHD in various genera (Ortiz-Masia et al., 2008; Xing et al., 2008; Pitzschke and Hirt, 2009; Zong et al., 2009; Liu and He, 2016).

In addition, studies have shown that more than 1000 lipids are engaged in stress-induced cell signaling, a number far exceeding that of structural lipids (Vigh et al., 2007a; van Meer et al., 2008). Lipids themselves appear to be versatile, acting not only as structural components but also as signaling mediators. One well-known example is the obvious rapid increases in PA and PIP_2_ observed under heat stress, the synthesis of which are catalyzed by PM-localized phospholipase D (PLD) and phosphatidylinositol-4-phosphate 5-kinase (PIPK), respectively (Mishkind et al., 2009; **Figure 1**). Heat-induced changes in membrane fluidity might activate phospholipases and kinases on the PM, including PLD and PIPK. Additionally, PIP_2_ is hydrolyzed by membrane-bound PLCs and releases diacylglycerol (DAG) and inositol 1,4,5-triphosphate (IP_3_), which increases rapidly within 3 min after heat shock in *Arabidopsis* (Zheng et al., 2012). IP_3_, which is generally considered a classic second messenger that is phosphorylated into IP_6_, is implicated in the regulation of intracellular calcium stores (Ren et al., 2017; **Figure 1**). However, an IP_6_ receptor has yet to be identified in plants. Furthermore, PIP_2_ itself also plays an important role in biotic and abiotic stress-signaling transduction that are related, for example, to membrane stability, the cytoskeletal order and the budding of secretory vesicles (Siddhanta and Shields, 1998; Di Paolo and De Camilli, 2006; Mishkind et al., 2009). PA is a structural lipid, and it acts as a key coordinator of the PLC signaling pathway in plants (Testerink and Munnik, 2005). *Arabidopsis* AtPLC3 and AtPLC9 are associated with the regulation of small *HSP* (*sHSP*) expression during the thermotolerance process by the same mechanisms (Zheng et al., 2012; Ren et al., 2017).

### Cold-Induced PM Stress Signal Cascades

A growth temperature that is higher or lower than the optimum range is defined as heat or cold stress. Although this concept is related to different plants, in general, cold-sensitive species can be harmed at temperatures below 15°C, while cold-tolerant plants may survive at temperatures close to 5°C (Chen, 1994; Nievola et al., 2017). As temperature effects, the cold signaling responses involve similar stages as those observed in plants exposed to heat. For instance, the cold first has an influence on the membrane fluidity via fatty acid saturation. In contrast to heat stress, low temperatures cause changes in the physical state of the membrane bilayer from a fluid-crystalline phase to a solid-gel phase, accompanied by decreased permeability, which is referred to as the membrane rigidity (Buchanan et al., 2015). Incremental membrane rigidity can also alter the activities of some channels, kinases and G-protein-associated receptors on the PM and thus rapidly induce increases in intracellular calcium, PA, and ROS similar to those in heat-treated cells (Plieth et al., 1999; Ruelland et al., 2002; Gupta et al., 2011). However, differences between these two types of temperature stress arise in the subsequent signal cascades (Kaplan et al., 2004; McClung and Davis, 2010; Zhou et al., 2014). The increased PA, which can be produced by activated PLD under cold conditions, can trigger ROS production in cells and may be involved in the ABA-dependent responses under cold stress (Ruelland et al., 2002; Guo, 2011; Gupta et al., 2011). In addition, a Ca^2+^ signature can be decoded by many calcium sensors (Miura and Furumoto, 2013), among which the CaM-binding transcription activator (CAMTA3) has been shown to bind to the promoters of C-repeat-binding factors (*CBF2/DREB1C*) and induce their gene expression (Doherty et al., 2009). The MAPK cascades are also implicated in the up-regulation of *CBF/DREB1s* transcription factors during cold stress signaling, including that of MPK3, MPK4, and MPK6 in *Arabidopsis* (Smékalová et al., 2014). CBF-dependent cold-responsive (*COR*) gene expression is considered to be a central regulatory pathway in the cold stress response (Chinnusamy et al., 2007), which is regulated by ICEs (inducers of *CBF* expression; Chinnusamy et al., 2003). The numbers and categories of *COR* genes are diverse; some of their products have been found to maintain cell membrane structures, activate ROS-scavenging enzymes, and promote the accumulation of osmolytes when plants are exposed to cold stress (Guy, 1990; Cook et al., 2004; Hannah et al., 2005; Maruyama et al., 2009). Interestingly, Fowler and Thomashow (2002) identified 130 *CORs* that were similarly induced by warm temperatures according to analyses in *CBF*-overexpressing plants. Furthermore, HSFC1 positively regulates *COR* gene expression independent of the CBF2 signaling pathway under freezing conditions (Park et al., 2015). Thus, the different types of temperature (heat or cold)-induced signal transduction share an affinity with each other in terms of both molecular components and signaling pathways, which may start at temperature-altered membrane fluidity and induce responsive gene expression to maintain cell integrity and function. Additionally, similar to studies on HSR, the specific plant sensors for the cold stress response and the mechanisms by which the sensors perceive cold signaling remain to be further elucidated (Miura and Furumoto, 2013).

## Roles of the Chloroplast and Mitochondrion in Heat Stress Sensing and Responses

**FIGURE 2:**
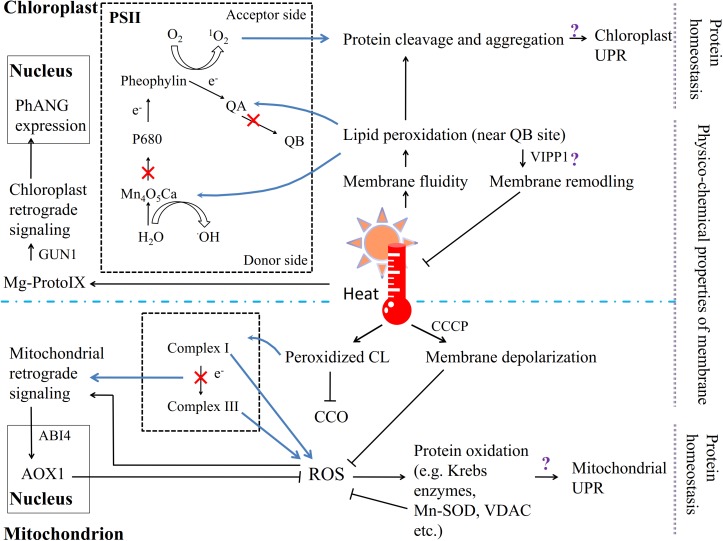
The chloroplast and mitochondrion are involved in the response to heat stress in plants. Except the PM, the other key sites of ROS generation are PSII in chloroplasts and complex I/III in mitochondria. Heat affects the fluidity of the thylakoid membrane, and unsaturated lipids near the QB site are particularly vulnerable to peroxidation. The lipid peroxidation products affect the stability of photosystem proteins; the electron transport chain is restrained, and ^1^O_2_ forms on the electron acceptor side and ^•^OH on the electron donor side. High temperatures trigger the chloroplast UPR and retrograde signaling pathway. Similarly, heat stress causes CL lipid peroxidation in the mitochondrion membrane, which inhibits the activity of CCO; but membrane depolarization via CCCP can inhibit ROS generation. The electron transport suppressed in the respiratory chain induces the production of ROS, which triggers both the mitochondrial UPR and retrograde signaling pathway.

The chloroplast and mitochondrion are membranous organelles in which carbon metabolism and energy exchange occur. Due to the ROS [including H_2_O_2_, superoxide anions (O_2_^∙-^), hydroxyl radicals (^•^OH), and singlet oxygen (^1^O_2_)] that are produced in the electron transport chain and the accompanying metabolic energy distribution during photosynthesis and respiration, chloroplast and mitochondrion components are the major targets of oxidative damage in plants, especially in the presence of other environmental stresses, such as high temperatures (Asada, 2006; Suzuki et al., 2012; Pospíšil and Prasad, 2014). Moreover, because both the chloroplast and mitochondrion are semiautonomous organelles regulated by the nucleus, the communication of organelle stresses to the nucleus and the modulation of the expression of stress-related genes, which are defined as retrograde signaling pathways, are considered to be extremely significant during physiological challenges, including heat stress (Sun and Guo, 2016; **Figure 2**).

### Chloroplasts

In chloroplasts, the photosynthetic apparatus primarily consists of the thylakoid membrane; all the components related to photon capture, electron transfer and energy exchange are found in different compartments of the thylakoid, and its lipid composition and physical state therefore appear to be particularly important for the photosynthetic system. Unlike the PM, the thylakoid membrane is composed of highly unsaturated glyceroglycolipids containing considerable amounts of monogalactosyldiacylglycerol (MGDG) and digalactosyldiacylglycerol (DGDG) as well as small amounts of phosphatidylglycerol (PG) and sulfoquinovosyldiacylglycerol (SQDG) (Dorne et al., 1990; Somerville et al., 2000; Pospíšil and Yamamoto, 2017). Hence, polyunsaturated fatty acids unavoidably become a site of attack by lipid peroxyl radicals under heat stress if the fatty acid moiety is near the Q_B_ site on the PSII electron acceptor side, suggesting that the fluidity of the thylakoid membrane is important for PSII function (Triantaphylides et al., 2008; Yamashita et al., 2008; Triantaphylides and Havaux, 2009; **Figure 2**). Moreover, both ^1^O_2_ formation on the electron acceptor side and ^•^OH production on the electron donor side result from the inactivation of PSII electron transport when plants are exposed to heat stress (Yadav and Pospíšil, 2012). For instance, high temperatures efficiently shift the midpoint redox potential of Q_A_/Q_A_^-^, which leads to the suppression of electron transfer from Q_A_ to Q_B_ on the electron acceptor side of PSII (Pospíšil and Tyystjärvi, 1999). In the green alga *Chlamydomonas reinhardtii*, ^1^O_2_ formation as initiated by heat-induced lipid peroxidation, in which lipoxygenase plays the primary role, has been demonstrated (Prasad et al., 2016). Lipid peroxidation products also have a pernicious effect on PSII proteins via oxidative modification, protein cleavage and irreversible protein aggregation (Chan et al., 2012; Yamamoto et al., 2014). Studies have shown that the reaction center binding protein D1 can be cleaved in spinach thylakoid membranes under heat stress (Yoshioka et al., 2006) and that lipid peroxidation occurs in spinach thylakoids (Yamamoto et al., 2008; Yamashita et al., 2008), and ^1^O_2_ is subsequently produced, which in turn causes D1 protein degradation via interactions with the protein (Pospíšil et al., 2007; Yamamoto et al., 2008; Yamashita et al., 2008; Pospíšil and Yamamoto, 2017). In addition, heat-induced lipid peroxidation can facilitate the denaturation and destabilization of PsbO (PsbP and PsbQ in spinach PSII) binding to the PSII core complex and promote manganese ion release from the Mn_4_O_5_Ca cluster, which has been demonstrated to be the main reason for the inactivation of electron transport on the donor side of PSII (Pospíšil et al., 2003; Yamauchi et al., 2008; Yamauchi and Sugimoto, 2010; Pospíšil and Yamamoto, 2017). In maintaining the chloroplast membrane under heat stress, a remarkable role is played by the vesicle-inducing protein in plastid 1 (VIPP1), which localizes to the chloroplast envelope and thylakoid membrane (Zhang et al., 2016). VIPP1 was recently indicated to be a GTPase involved in maintaining the integrity of photosynthetic membranes via an effect on membrane remodeling when plants were subjected to stresses (Ohnishi et al., 2018).

Chloroplast retrograde signaling can be initiated by the accumulation of the chlorophyll intermediate Mg-protoporphyrin IX (Mg-ProtoIX, belonging to tetrapyrroles), as shown by *gun* (genome uncoupled) mutants, which exhibit impaired communication from the chloroplast to the nucleus, controlling photosynthesis-associated nuclear gene (*PhANG*) expression (Susek et al., 1993; Mochizuki et al., 2001; Larkin et al., 2003; Strand et al., 2003; Koussevitzky et al., 2007; Woodson and Chory, 2008; Barajas-López Jde et al., 2013; Zhang et al., 2015; **Figure 2**). *GUN1*, which encodes a unique plastid-localized pentatricopeptide repeat (PPR) protein, has been shown to be required to transmit stress signaling to the nuclear transcription factor ABA insensitive 4 (ABI4; Koussevitzky et al., 2007). Additionally, ABI4 is considered to be a central regulator that is involved in many essential environmental signaling pathways and in the developmental as well as central metabolic processes of cells or organelles (reviewed by León et al., 2013). Findings in *Chlamydomonas reinhardtii* have further indicated that Mg-ProtoIX transiently promotes the expression of heat shock protein 70 (HSP70), which may localize to both the plastid and cytoplasm (Kropat et al., 1997, 2000). In addition, nuclear gene transcriptional studies on the above green alga showed that more than half of tetrapyrrole-interrelated genes were also activated by heat stress (Voss et al., 2011). Moreover, the chloroplast ribosomal protein S1 (RPS1) was reported to activate the expression of *HSFA2*, which is considered to regulate heat-stress-responsive genes during heat acclimation (Yu et al., 2012; Liu and Charng, 2013).

### Mitochondria

In the mitochondria, a type of phospholipid known as cardiolipin (CL), which has a high percentage of polyunsaturated fatty acids, is embedded in the mitochondrial membrane, where lipid peroxidation occurs (Bligny and Douce, 1980; Caiveau et al., 2001; **Figure 2**). Damaged CL strongly decreases the activity of cytochrome c oxidase (CCO) under stress (Paradies et al., 1998). Moreover, in *A. thaliana*, cardiolipin synthase (CLS) is required for mitochondrial morphogenesis and the response to high-temperature stress (Pan et al., 2014). The physical state of the mitochondrial membrane is also crucial; the depolarization of the mitochondrial membrane by the protonophore CCCP has been shown to inhibit ROS generation under thermal treatment (Fedyaeva et al., 2014). Additionally, the mitochondrion is one of the key compartments in ROS generation, especially when the rate of the electron transport chain has been decreased by environmental stresses (Møller and Kristensen, 2004). ROS negatively affect both lipids and proteins. The products of protein oxidation consist mostly of tryptophan (Taylor et al., 2003) and products of carbonylation and fragmentation (Sweetlove et al., 2002). For example, some Krebs cycle enzymes, other redox enzymes, and HSPs as well as Mn superoxide dismutase (Mn-SOD) and the voltage-dependent anion channel from rice leaf mitochondria, can be carbonylated under stress (Kristensen et al., 2004; **Figure 2**).

The mitochondrial retrograde signaling pathway also shows a relationship with ROS generation and accumulation (Rhoads et al., 2006; Rhoads and Subbaiah, 2007; **Figure 2**). When electron transport is disrupted by abiotic stresses, the increased expression of nuclear-encoded alternative oxidase 1 (*AOX1*) regulates the ROS level to trigger retrograde responses in plants. The *AOX1A* promoter sequence is one of the targets of ABI4 in *A. thaliana*; ABI4 is involved in mitochondrion-to-nucleus retrograde signaling as well as that in chloroplasts (Giraud et al., 2009). Because mitochondrial complexes I and III are the primary sites of ROS generation, a mutation in complex I can promote ROS accumulation and decrease the expression of *COR* genes (Lee et al., 2002; Møller and Kristensen, 2004). Mitochondrion-derived DEXH box RNA helicases or PPR proteins also induce ROS accumulation and are involved in ABA responses (He et al., 2012).

### Calcium Ions and HSPs in Chloroplasts and Mitochondria

The roles of Ca^2+^ and HSPs in the regulation of organelle signaling under high-temperature stress should not be ignored. Earlier studies showed that chloroplasts contained high total Ca^2+^concentrations (Brand and Becker, 1984; Evans et al., 1991), while the potential of the plant inner mitochondrial membrane may affect the level of cytosolic Ca^2+^ and, thus, the expression of heat-responsive genes (Pyatrikas et al., 2014; Rikhvanov et al., 2014). However, further studies are needed in this field.

The HSPs exhibit a chaperone function, folding polypeptides into a mature state or preventing misfolded proteins from aggregation, and they have been found to accumulate in cells under thermal stress (Trösch et al., 2015). Because most proteins in chloroplasts and mitochondria are encoded by nuclear genes, and the polypeptides are exported from the nucleus to the organelles, HSPs are correspondingly required for the protein conformational changes during these processes. HSP70 acts upstream of other HSPs and cooperates with other chaperones in protein folding, refolding and disaggregation (Langer et al., 1992; Haslberger et al., 2007). Chloroplast stromal HSP70 was found to be involved in PSII repair under stress and to be affected by the redox state of the chloroplast (Schroda et al., 1999; Liu et al., 2007b; Rutgers and Schroda, 2013; Trösch et al., 2015). Another HSP, HSP90, more likely serves as a regulator of signal transfer to the nucleus, including the modulation of photosynthesis-related genes and stress-related genes (Beck, 2005; Trösch et al., 2015), although it can also stabilize unfolded proteins (Nathan et al., 1997). The sHSPs are chaperones that show high diversity among species and tissues and can prevent improper protein aggregation and partially unfold under stress conditions (Bernfur et al., 2017). Several lines of evidence have suggested that a small chloroplast HSP, HSP21, is involved in plant adaptations to heat stress and oxidative stress (Heckathorn et al., 1998; Shakeel et al., 2011; Kim et al., 2012). A recent study demonstrated that HSP21 could bind to and stabilize PSII core proteins D1 and D2 under heat stress, which was activated by the GUN5-associated retrograde signaling pathway (Chen et al., 2017). *GUN5* encodes an Mg chelatase that can insert Mg^2+^ into the protoporphyrin ring during tetrapyrrole biosynthesis, which regulates ROS-induced gene expression (Mochizuki et al., 2001; Adhikari et al., 2011). Furthermore, HSP21 was demonstrated to interact with another protein, plastid transcriptionally active 5 (pTAC5), which is required for plastid-encoded RNA polymerase (PEP)-dependent transcription, and the latter protein plays a crucial role in plastid development under heat stress (Zhong et al., 2013).

## ER Stress and the Unfolded Protein Response are Involved in Plant Adaptive Responses to High Temperatures

### ER Stress

The ER is the largest endomembrane system, and it is responsible for protein synthesis, folding, modification and export as well as lipid metabolism. More than a third of the total proteins pass through the secretory pathway via the ER lumen (Ron and Walter, 2007; Fragkostefanakis et al., 2016). Therefore, ER homeostasis is highly susceptible to environmental cues, inevitably resulting in the production of misfolded proteins or the accumulation of unfolded proteins, which are also known as ER stress (Walter and Ron, 2011). The mechanism underlying the response to ER stress at the transcriptional and translational levels is referred to as the unfolded protein response (UPR), which includes the detection of unfolded or misfolded proteins; the activation of chaperones, foldases and pertinent factors; and the reduction of the secreted and membrane protein contents. If the UPR is overextended and ER homeostasis cannot be sustained, cells will initiate programmed death (Deng et al., 2013). In addition to aberrant proteins, instability of phospholipid metabolism also leads to ER stress and activates the UPR (Yu et al., 2018). Studies on yeast and mammalian cells revealed that an imbalanced PC to PE ratio is a crucial cause of ER stress (Fu et al., 2011; Thibault et al., 2012; Gao et al., 2015). However, the link between lipid functions and the ER stress response is still poorly understood in plants. Yu et al. (2018) verified that *Arabidopsis* glycerolipid homeostasis might be sustained at the post-transcriptional level under ER stress. Additionally, a study on maize illustrated that ER stress could affect the enzyme activity of diacylglycerol kinase (DGA), choline phosphate cytidylyltransferase, phosphatidylinositol-4-kinase, and PIPK (Shank et al., 2001).

### ER Stress Sensors Perceive High-Temperature Stress

Nascent proteins require molecular chaperones to prevent their aggregation and assist in their folding into functional conformations. Binding protein (BiP), an HSP70, is a common chaperone in the ER that is recruited by the DNAJ protein ERdj3 to bind unfolded proteins (Jin et al., 2009; **Figure 1**). Other chaperones known as foldases have also been described in several previous reviews (Gupta and Tuteja, 2011; Deng et al., 2013).

Plants harbor two primary signaling pathways that have been reported to sense ER stress. One is aided by membrane-associated transcription factors (the basic leucine zipper transcription factors bZIP17 and bZIP28), and the other involves an RNA splicing factor (the inositol-requiring enzyme IRE1) (Liu and Howell, 2016; **Figure 1**). The bZIP28 can specifically sense heat-triggered ER stress via BiP, after which it translocated into the Golgi, and its TF domain on the cytosol side can be cleaved by proteolytic processing. The TF portion subsequently enters the nucleus to activate the expression of stress-related genes. In addition, IRE1 is activated by interaction with unfolded proteins and then recognizes and splices the mRNA of *bZIP60*, after which the splicing products enter the nucleus to trigger the expression of UPR genes (Deng et al., 2011; Liu and Howell, 2016; **Figure 1**). Heat stress has been validated as a direct and efficient factor that influences the nuclear relocation of bZIP28 and the mRNA splicing of *bZIP60* (Che et al., 2010; Deng et al., 2011).

## Conclusion and Perspective

Temperature fluctuation is an unusual ambient signal that causes concurrent alterations in multiple cellular compartments, including the PM, cytosol, energy-associated organelles and chromatin (Zhu, 2016). In fact, there is widespread support for the notion that an increase in PM fluidity as induced by high temperatures first activates stress-signaling pathways (reviewed by Ruelland and Zachowski, 2010). However, how should the concept of “primary heat sensors” be understood? If the macromolecules in plant cells are defined as thermosensors that are able to not only precisely perceive various temperature changes but also differentially trigger specific signaling pathways (Mittler et al., 2012), the most feasible strategy might be to first divert ambient temperature signals into different cell signaling patterns before they are perceived by sensors. From this perspective, the specific role of the membrane might be to serve as both a thermometer and a stress encoder. Additionally, subtle changes in the lipid composition of fatty acid chains or head groups can alter both the membrane properties and the interaction with specific proteins and even the membrane substructures (microdomains), which are highly sensitive to environmental perturbation (Barrero-Sicilia et al., 2017); in this context, the membrane clearly meets the requirement for encoding stress. Of course, more evidence must be obtained and evaluated to dissect the mechanisms of membrane-associated stress sensing and signaling. One important reason why it is so difficult to study these mechanisms is that membrane structures in plant cells possessing a cell wall exhibit an enormous variety of membrane components with diverse localizations, interactions, and functions (Escribá et al., 2008; Malinsky et al., 2013). The conventional methods for the investigation of membrane proteins have obvious limitations (e.g., specific protein associations can be disrupted by detergents; Tanner et al., 2011; Malinsky et al., 2013). The other reason is that the available studies on lipids and relevant enzymes are not equivalent to the attention paid to the proteins involved in signal transduction (Eyster, 2007), despite the fact that membrane lipids show the best fit for the response to various environmental cues among these molecules (Escribá et al., 2008). Fortunately, the direct visualization of the distribution of membrane constituents that rely on fluorescence microscopy approaches makes it possible to detect the dynamic interaction and distribution of PM substructures (reviewed by Malinsky et al., 2013; Konrad and Ott, 2015; Wang et al., 2018). In addition, based on improvements in the sensitivity and accuracy of mass spectrometry and chromatography as well as microarray transcriptome analyses, it is possible to obtain knowledge of the entire lipidome and the complicated regulatory network at a certain time point in an individual tissue type under stresses (Cacas et al., 2012; Higashi et al., 2015; Marla et al., 2017).

Furthermore, numerous vesicles in cells are also an important part of the membrane system whose formation and trafficking in plants have been shown to be involved in membrane repair under abiotic and biotic stresses (reviewed by Hadlington and Denecke, 2000; Frei and Robatzek, 2009; Baral et al., 2015). However, much about the underlying mechanism of communication between extra-membrane and membranous organelles remains to be elucidated. Moreover, heat stress is inevitably associated with various other stresses during actual crop production. For example, high temperatures are often accompanied by high light, which can accelerate the plant injury inflicted by drought and salinity stress in inland regions. Elevated temperatures have also been shown to promote the infection of plant cells by pathogens and cause damage to SA defense (Huot et al., 2017). The identification and elucidation of these “crosstalk” mechanisms seem precisely the task of the day.

In short, although studies on the underlying mechanisms of plant adaptation to high temperature and the engineering of crop plants with higher thermotolerance are making steady progress, many questions are still open and need to be further addressed. Important issues include the following:

(i) Are other primary thermosensors present in the PM or other intracellular compartments that synergistically trigger multiple and/or specific responses?
(ii) How is the signaling that is triggered by various forms, durations and intensities of high-temperature stress transmitted?
(iii) What precise role do membrane microdomains play in the plant HR?
(iv) How can the dynamic remodeling of membranes under various heat stresses be recorded or mimicked?
(v) How do second messengers transmit multiple signals to downstream components of HS signal transduction chains?
(vi) How do the UPR in chloroplasts, the cytosol and the ER coordinately and collaboratively function in protein homeostasis?
(vii) How does the PM contact other membranous organelles under elevated temperatures?
(viii) How does the lipid metabolism in chloroplasts and the ER participate in membrane sensing and remodeling during the rapid HR?
(ix) Apart from HSF-HSP responses, are there other HSR effectors that switch the comprehensive responses on/off in plant cells?
(x) How is heat stress sensing distinguished from and integrated with the sensing of other stresses?

## Author Contributions

YN prepared and wrote this manuscript. YX contributed by organizing and reviewing a major part of the manuscript.

## Conflict of Interest Statement

The authors declare that the research was conducted in the absence of any commercial or financial relationships that could be construed as a potential conflict of interest.
